# Hepcidin overexpression in astrocytes alters brain iron metabolism and protects against amyloid-β induced brain damage in mice

**DOI:** 10.1038/s41420-020-00346-3

**Published:** 2020-10-30

**Authors:** Xinwei Zhang, Yu-Jing Gou, Yating Zhang, Jie Li, Kang Han, Yong Xu, Haiyan Li, Lin-Hao You, Peng Yu, Yan-Zhong Chang, Guofen Gao

**Affiliations:** 1 Laboratory of Molecular Iron Metabolism, College of Life Sciences, Hebei Normal University, No. 20, Nan Er Huan East Road, 050024 Shijiazhuang, China; 2 Chengde Medical University, Shuang Qiao District, An Yuan Road, 067000 Chengde, China

## Abstract

Progressive iron accumulation in the brain and iron-induced oxidative stress are considered to be one of the initial causes of Alzheimer’s disease (AD), and modulation of brain iron level shows promise for its treatment. Hepcidin expressed by astrocytes has been speculated to regulate iron transport across the blood–brain barrier (BBB) and control the whole brain iron load. Whether increasing the expression of astrocyte hepcidin can reduce brain iron level and relieve AD symptoms has yet to be studied. Here, we overexpressed hepcidin in astrocytes of the mouse brain and challenged the mice with amyloid-β_25–35_ (Aβ_25–35_) by intracerebroventricular injection. Our results revealed that hepcidin overexpression in astrocytes significantly ameliorated Aβ_25–35_-induced cell damage in both the cerebral cortex and hippocampus. This protective role was also attested by behavioral tests of the mice. Our data further demonstrated that astrocyte-overexpressed hepcidin could decrease brain iron level, possibly by acting on ferroportin 1 (FPN1) on the brain microvascular endothelial cells (BMVECs), which in turn reduced Aβ_25–35_-induced oxidative stress and apoptosis, and ultimately protected cells from damage. This study provided in vivo evidences of the important role of astrocyte hepcidin in the regulation of brain iron metabolism and protection against Aβ-induced cortical and hippocampal damages and implied its potential in the treatment of oxidative stress-related brain disorders.

## Introduction

Iron plays vital roles in various physiological activities in the brain, including oxygen transport, DNA synthesis, mitochondrial respiration, myelination, neurotransmitter synthesis, and so on^1,2^. However, through the Fenton reaction, excessive iron can induce the generation of reactive oxygen species (ROS) that is damaging to the cells^1,3^.

Accumulating evidences from human and animal models have indicated a close association between iron dysregulation and the pathogenesis of Alzheimer’s disease (AD)^4,5^. It has been demonstrated that iron heavily accumulated in amyloid-β (Aβ) plaques and neurofibrillary tangles of the AD brain^5–7^, where it participated in redox cycling and led to oxidative damage^4,8,9^. In addition, the expressions of iron transporters and iron regulatory proteins were dysregulated in AD^10–13^. Modulation of the brain iron level or its metabolism to maintain brain iron homeostasis has been considered to be a promising therapy for AD^4,14–16^.

Hepcidin is the major regulator of systemic iron homeostasis^17^. Hepcidin binds to the iron exporter ferroportin 1 (FPN1) on the cell membrane, leading to FPN1 degradation and resulting in the reduction of iron release from the target cells^18^. In the peripheral system, hepcidin secreted by hepatocytes strictly regulates the transport of iron in intestinal and duodenal cells, thus maintaining the balance of the systemic iron level^19,20^. In the brain, hepcidin is widely distributed in neurons, glial cells, and vascular endothelia at blood–brain barrier (BBB) in various regions^21–23^. It has been reported that hepcidin expression was significantly reduced in hippocampi of AD patients and mouse models along with iron deposition^24^. However, the specific role of hepcidin in AD and targeting of hepcidin for AD treatment have not been reported.

Great progress has been made to understand the role of hepcidin in brain iron metabolism^25^. Injection of hepcidin expression adenovirus significantly reduced brain iron in rats and suppressed transport of transferrin-bound iron from the periphery into the brain^26^. Studies have also shown that hepcidin could protect neurons from hemin-mediated injury^27^, and the hepcidin level was closely associated with neurological inflammatory responses^28,29^. Besides, the expression amount and regional distribution patterns of hepcidin were age-dependent^30^. These findings suggested that a high level of hepcidin could reduce iron overload in the brain, which might be a potentially valuable target for AD treatment. Moreover, it has been proposed that hepcidin secreted by astrocytes could regulate FPN1 on brain microvascular endothelial cells (BMVECs), inducing the internalization and degradation of FPN1, and thus control iron transport across the BBB^31–33^. Therefore, increasing the expression of hepcidin in astrocytes might reduce brain iron intake and provide a good strategy for reducing the deposition of brain iron and alleviating the symptoms of AD.

In this study, we pre-overexpressed hepcidin in astrocytes of mouse brain and then challenged mice with Aβ_25–35_ peptide, a peptide widely used to induce AD symptoms in mice and rats^34–36^. The effects of hepcidin overexpression in astrocytes on Aβ_25–35_-induced damage in the cortex and hippocampus of the mouse brain were investigated. We demonstrated for the first time with in vivo evidences that astrocyte hepcidin could modulate brain iron metabolism and prevent iron overload in the brains of Aβ-treated mice, and therefore exhibit a protective effect against Aβ_25–35_-induced oxidative damage and apoptosis.

## Results

### Astrocyte-specific overexpression of hepcidin protected against Aβ-induced cognitive decline in mice

**Fig. 1:**
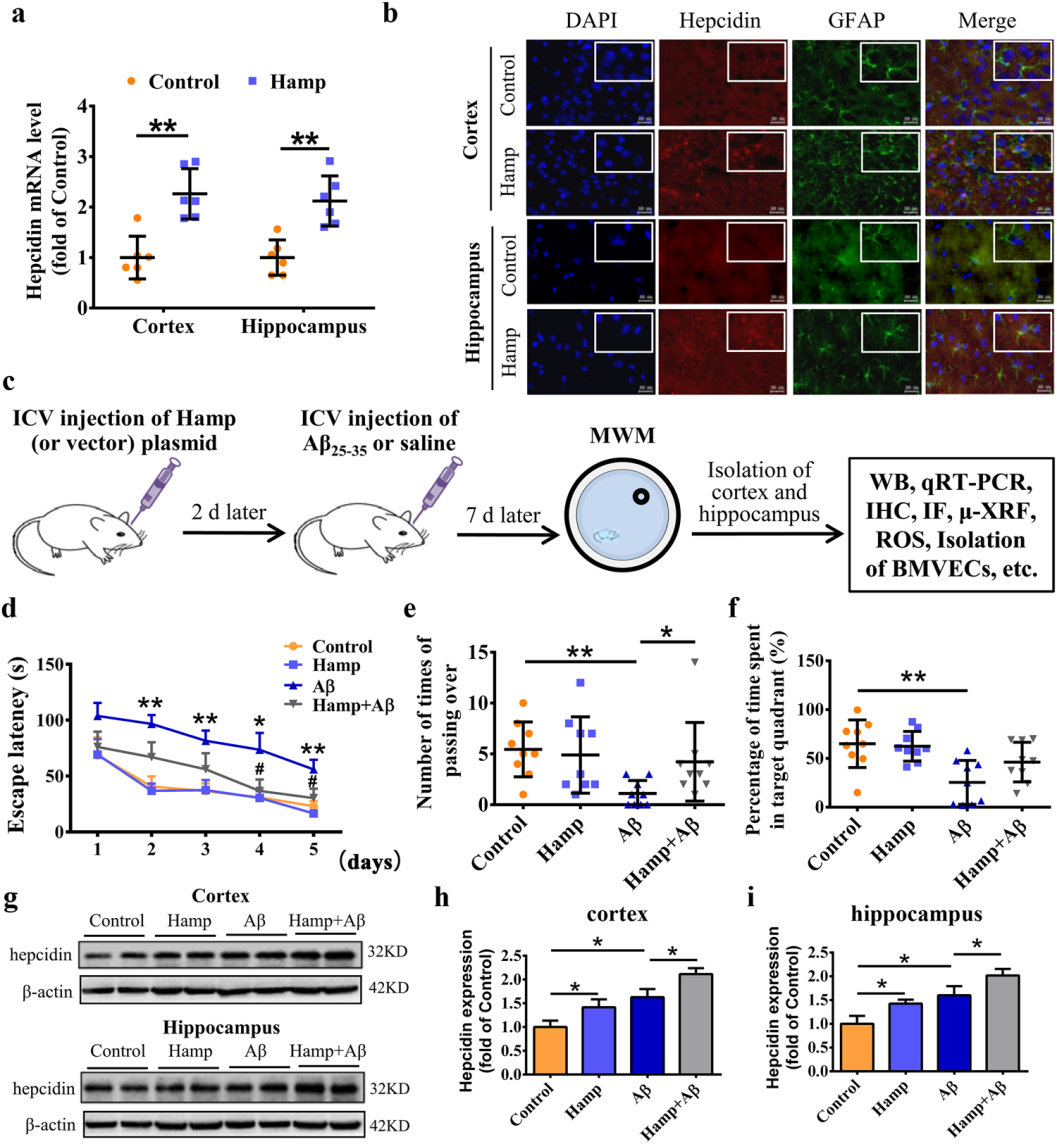
Astrocyte-specific overexpression of hepcidin protected against Aβ-induced cognitive decline in mice. **a** Quantifications of hepcidin mRNA in the cortex and hippocampus of mouse brains by qRT-PCR after 9 days of intracerebroventricular (ICV) injection. Data are presented as the means ± SD, *n* = 6. *P < 0.01 vs. the vector-injected control mice (Control). **b** Hepcidin (red) and GFAP (astrocyte marker, green) immunofluorescence staining were performed in the cortex and hippocampus near the lateral ventricle. DAPI (blue) was used for nuclear staining. Bar = 50 μm. **c** Experimental scheme: mice were ICV-injected with Hamp plasmid or vector control, and 2 days later injected with Aβ_25–35_ or saline. The behavior performance was assessed by MWM after 7 days, and then the cortical and hippocampal tissues were excised and analyzed by various methods, as indicated. **d** Data show the changes in latency time to find the hidden platform over the 5 consecutive days of training in MWM test. Data are presented as the means ± SEM, *n* = 9. *P < 0.05 and **P < 0.01 vs. the control group; ^#^*P* < 0.05 vs. the Aβ-treated group. **e**, **f** The number of times the mouse traversed the platform (**e**) and the percentage of time spent in the target quadrant (**f**) on the 6th day after removing the platform. Data are presented as the means ± SD, *n* = 9. *P < 0.05 and **P < 0.01. **g** Representative western blots images for detection of the hepcidin expression in the cortex and hippocampus of different mice. **h**, **i** The relative expression level of hepcidin in the cortex (**h**) and hippocampus (**i**) were calculated after normalizing each specific band to its respective β-actin band, and are presented as means ± SEM, *n* = 6. *P < 0.05.

To overexpress hepcidin in astrocytes of mouse brain, we constructed a pAAV-gfap:*Hamp* plasmid with mouse hepcidin gene (*Hamp*) under the control of astrocyte-specific GFAP promoter. To confirm its expression efficacy and astrocyte specificity, mice were administrated with this plasmid or the vector control by intracerebroventricular (ICV) injection. qRT-PCR analysis revealed increased hepcidin mRNA levels in the cerebral cortex and hippocampus of mice injected with *Hamp* plasmid, and immunofluorescence staining indicated good specificity for astrocytes (Fig. 1a, b).

To investigate the role of astrocyte hepcidin overexpression in Aβ-induced brain damage, we injected mice with Aβ_25–35_ peptide after hepcidin overexpression^35,36^. The spatial learning and memory abilities of mice in different groups were assessed by MWM test after 1 week (Fig. 1c). Compared with the mice without Aβ treatment (control group), Aβ-treated mice (Aβ group) displayed a significant learning and memory decline, as indicated by the longer escape latency time during training (Fig. 1d), the lower number of times passing over the target zone (Fig. 1e), and the shorter time spent in the target quadrant after removing the platform (Fig. 1f). Strikingly, the mice pre-injected with *Hamp* and then injured by Aβ (Hamp + Aβ group) showed significantly improved learning and memory abilities compared with the mice in Aβ group. The Hamp + Aβ mice exhibited better escape latency to the platform at day 4 and day 5 compared with Aβ mice (Fig. 1d, indicated by #), and also crossed the platform area more times after removing the platform (Fig. 1e). The hepcidin expression in the brain of different groups of mice was assessed by western blot. As shown in Fig. 1g–i, the Hamp and Hamp + Aβ groups showed significantly higher levels of hepcidin than the control and Aβ groups, respectively, in both the cortex and hippocampus regions. The Aβ group showed a higher level of hepcidin than the control group, indicating that the Aβ_25–35_ peptide triggered inflammatory responses and induced hepcidin expression^19^.

In addition, we examined the levels of postsynaptic density protein-95 (PSD-95) in the hippocampus of different mice^37^. The results showed that PSD-95 mRNA and protein expression was reduced in the Aβ group compared to the control group, while astrocyte hepcidin overexpression rescued this reduction (Supplementary Fig. S1). These results suggest that the overexpression of hepcidin in astrocytes significantly alleviates learning and memory deficits in the Aβ-induced AD model.

### Astrocyte-specific overexpression of hepcidin alleviated Aβ-induced apoptosis

**Fig. 2:**
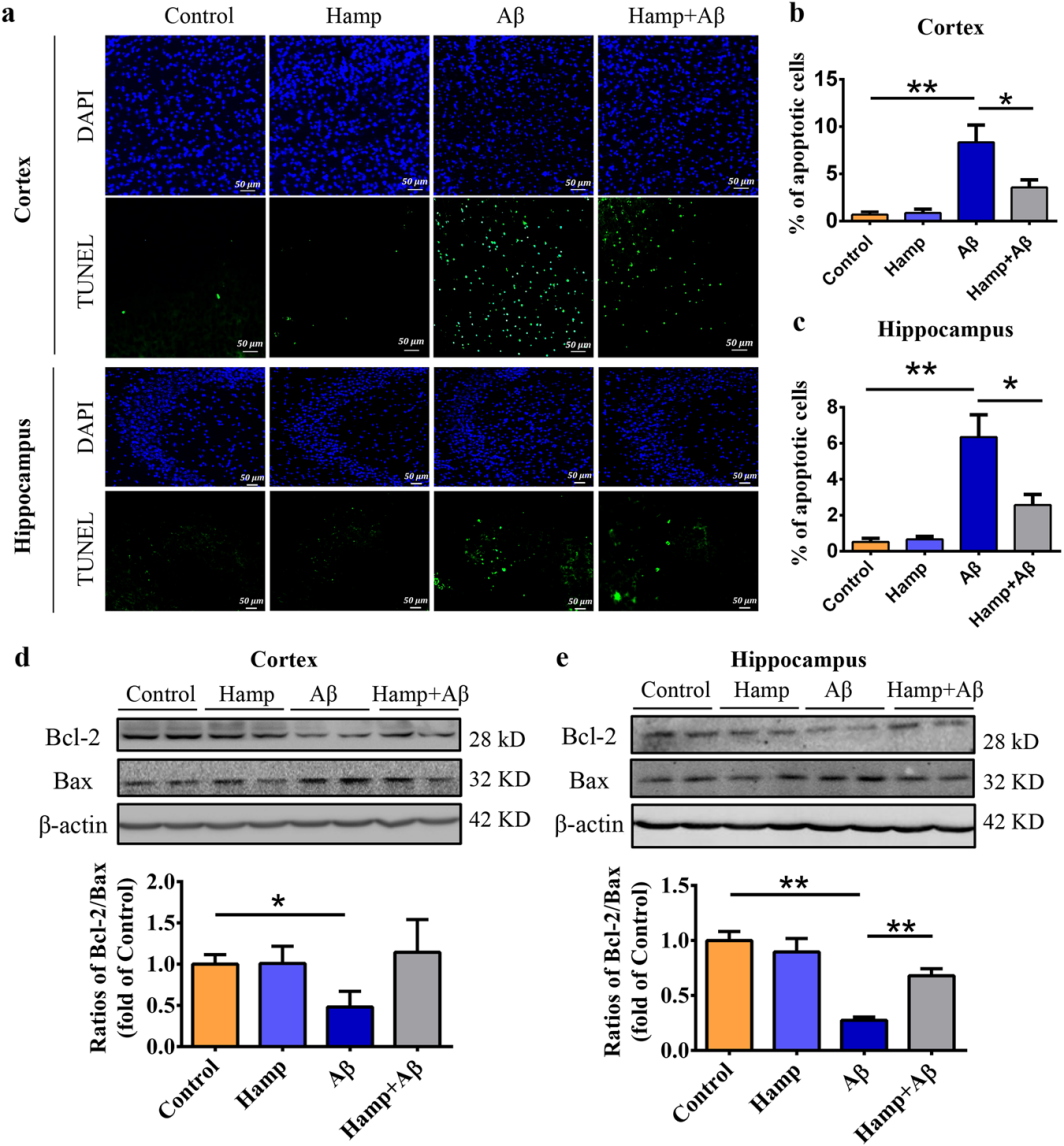
Astrocyte-overexpressed hepcidin alleviated Aβ-induced apoptosis in the mouse brain. **a** TUNEL-positive cells (green) and DAPI-stained nuclei in the cortex near lateral ventricles and hippocampal CA3 regions of mice in different groups were observed. Bar = 50 μm. **b**, **c** The statistical analysis of relative apoptotic cell levels in cortical sections (**b**) and hippocampal sections (**c**). Data are presented as the means ± SEM, *n* = 6. **d**, **e** Western blot was used to detect expression levels of Bcl-2 and Bax in the cortex (**d**) and hippocampus (**e**) in different groups of mice, and the ratios of Bcl-2/Bax were calculated and expressed as fold of control. Data are presented as the means ± SEM, *n* = 6. *P < 0.05 and **P < 0.01.

The trigger of apoptosis by Aβ_25–35_ peptides was reported previously^36^. The damage induced by Aβ_25–35_ was specific as compared to Aβ_35–25_ control peptide, and the apoptotic cells included both neurons and glial cells (Supplementary Fig. S2). We assessed the apoptotic levels in the cortex and hippocampus of different mice by TUNEL staining. It was found that Aβ_25–35_ treatment induced a large number of apoptotic cells in the cortex and CA3 region of the hippocampus (Fig. 2a–c). Interestingly, overexpression of hepcidin in astrocytes and subsequent administration of Aβ_25–35_ resulted in a visible decrease in apoptotic cells in both the cortex and hippocampus (Fig. 2a–c). The contents of Bcl-2 (anti-apoptotic protein) and Bax (pro-apoptotic protein) in the cortical and hippocampal tissues were also quantified to reflect the apoptosis status of different groups^38^. As shown in Fig. 2d, e, a dramatic Bcl-2 inhibition and Bax activation were observed in the cortex and hippocampus of Aβ-treated mice compared with the control mice, resulting in significantly reduced Bcl-2/Bax ratios. In contrast, the Hamp + Aβ mice with hepcidin overexpression in astrocytes maintained the levels of both Bcl-2 and Bax, showing a relatively small change in Bcl-2/Bax ratios (Fig. 2d, e). These indicate that the elevated astrocyte hepcidin can partially alleviate Aβ_25–35_-induced apoptosis in the cortex and hippocampus of the mouse brain.

### Astrocyte hepcidin overexpression reduced Aβ-induced oxidative damage

**Fig. 3:**
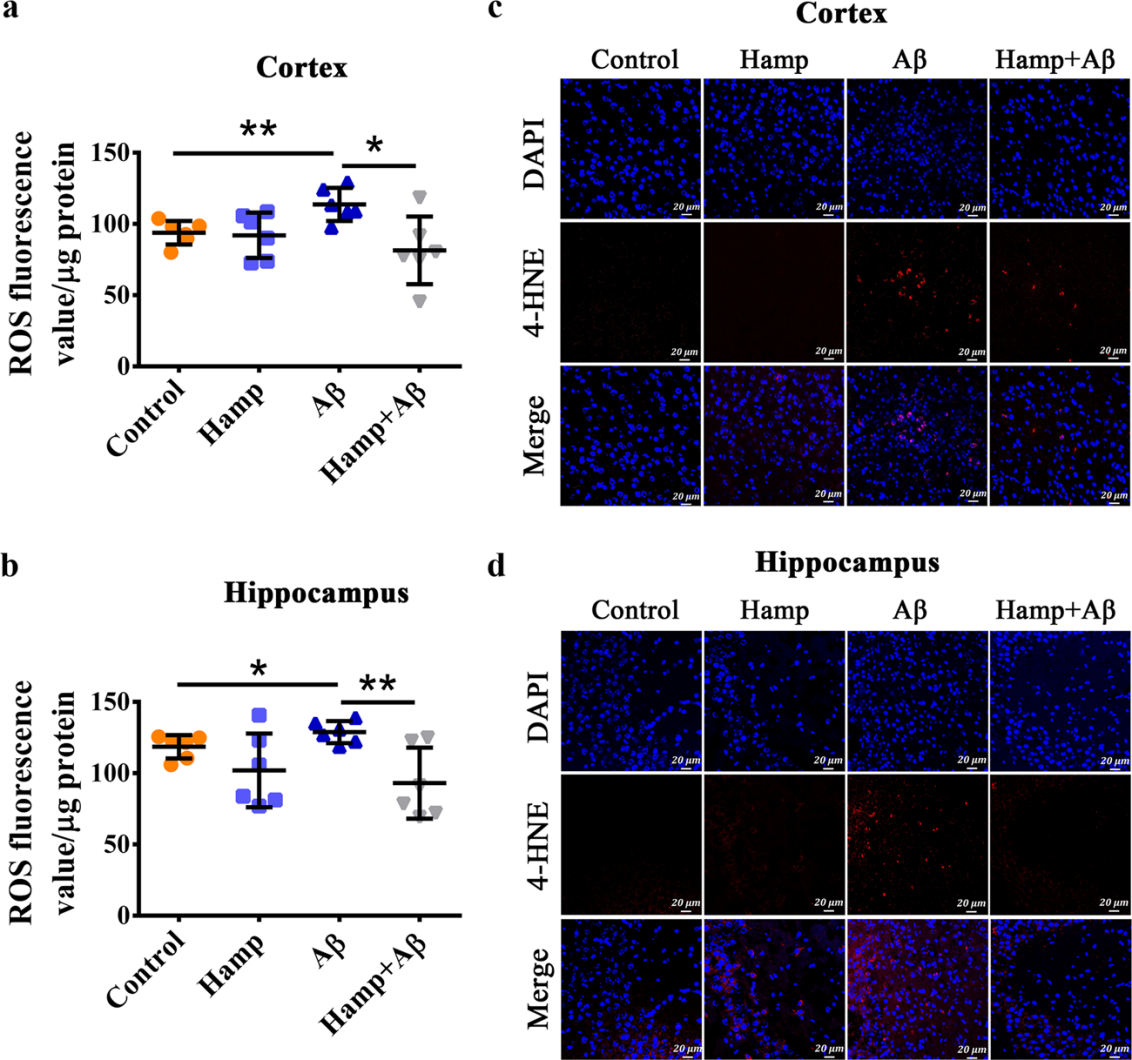
Astrocyte hepcidin reduced oxidative damage. **a**, **b** Reactive oxygen species (ROS) levels of the cortex (**a**) and hippocampus (**b**) were assayed as described in the “Materials and methods”. Data are presented as the means ± SD. *n* = 6. *P < 0.05 and **P < 0.01. **c**, **d** Immunofluorescence staining of 4-HNE (red) was carried out in sections of the cortex (**c**) and hippocampus (**d**). DAPI was used for nuclear staining. Bar = 20 μm.

Since apoptosis is usually associated with oxidative stress, we examined the levels of ROS and 4-HNE, a byproduct of lipid peroxidation during oxidative stress^8^. As shown in Fig. 3a, ROS generation in the cortex of Aβ-treated mice had a substantial increment, whereas it decreased significantly in Hamp + Aβ mice. Similarly, ROS level in the hippocampus of Hamp + Aβ mice was also lower than that of Aβ-treated mice (Fig. 3b). The level of 4-HNE increased largely in Aβ_25–35_-injected mice compared with controls, but it reduced significantly in both the cortex and hippocampus of Hamp + Aβ mice (Fig. 3c, d). These demonstrate that the astrocyte-overexpressed hepcidin indeed reduces oxidative stress induced by Aβ.

It is well known that the MAPK pathway participates in the signaling cascade of cellular stress responses^39^, and is closely related to the pathogenesis of AD^40^. This signaling pathway is further capable of modulating mitochondria-mediated apoptosis in the Bcl-2 family^41,42^. Therefore, we explored the activation of the MAPK/ERK/p38 pathway in mice of different groups. As shown in Supplementary Fig. S3, the phosphorylation of both p38 and ERK was largely increased in the cortex and hippocampus of Aβ_25–35_-treated mice, but much less in Hamp + Aβ mice.

### Astrocyte hepcidin overexpression prevented Aβ-induced brain iron overload

**Fig. 4:**
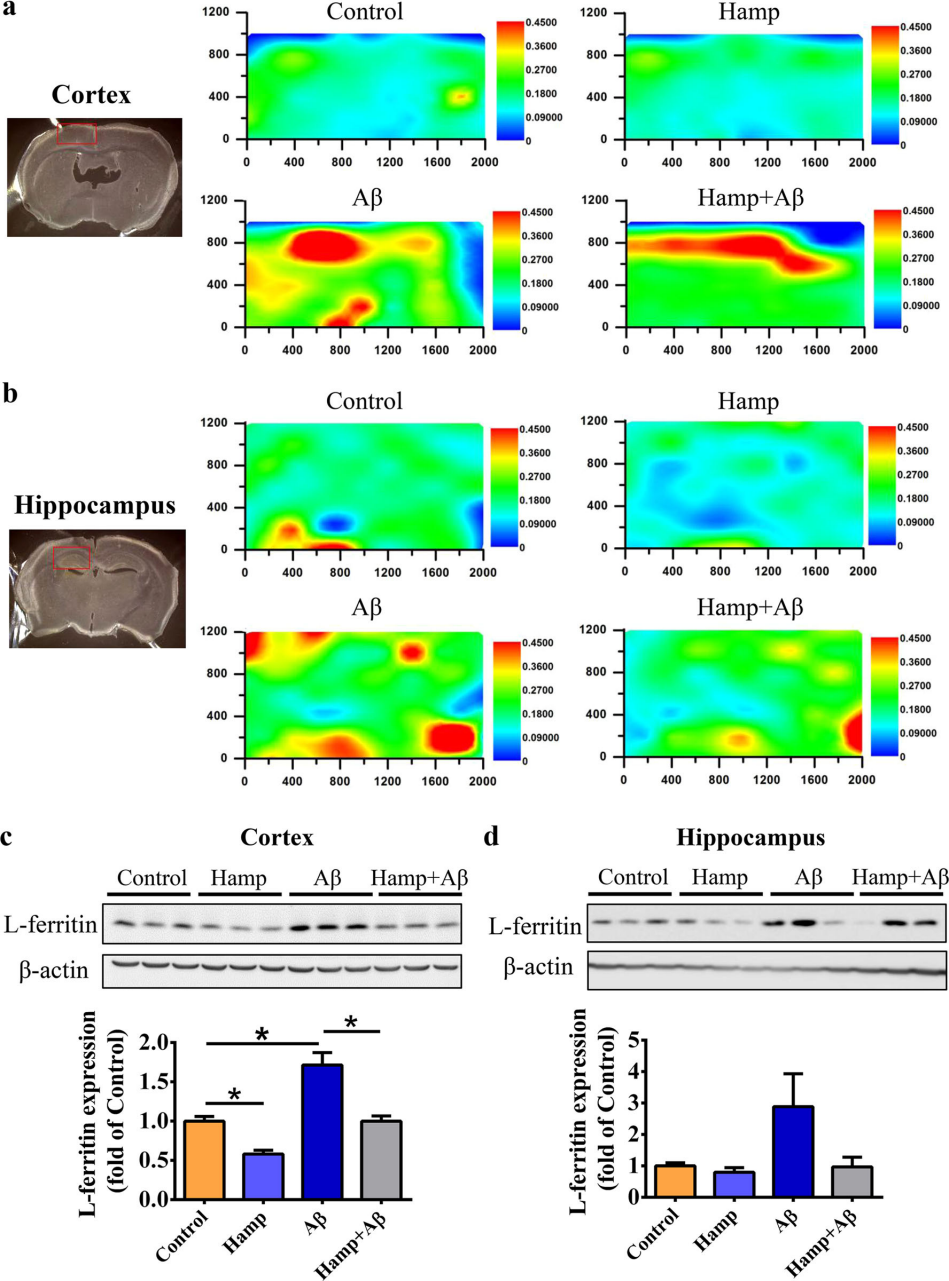
Astrocyte hepcidin ameliorated Aβ-induced elevation in iron levels. **a**, **b** Detection of brain iron distribution by synchrotron radiation μ-XRF in the cortex (**a**) and hippocampus (**b**). The iron content in the blue region is the lowest, and increases gradually from blue to red, while the iron content in the red region is the highest. **c**, **d** Western blot was used to measure L-ferritin in the cortex (**c**) and hippocampus (**d**) of mice in different groups. The relative expression levels in different groups as compared to the control group were calculated after normalizing each specific band to its respective β-actin band, and are presented as means ± SEM, *n* = 6. *P < 0.05 and **P < 0.01.

It is notable that excess free iron contributes to the generation of ROS and oxidative stress^4,43^. We, therefore, investigated whether Aβ_25–35_ induces elevation of brain iron and whether the overexpression of hepcidin in astrocytes prevents iron overload and subsequently reduced oxidative stress and apoptosis. To examine iron levels, we utilized μ-XRF to directly visualize iron distribution in the cortex and hippocampus of mice in different groups. Compared with the control group, the iron content in the cortex and hippocampus of Aβ-treated mice increased dramatically (Fig. 4a, b), whereas the iron content of Hamp + Aβ mice indeed reduced as compared to the Aβ-treated mice, although still higher than that of the control group (Fig. 4a, b). To further confirm the changes of iron level, we measured the expression of iron storage protein, L-ferritin. Consistent with the change of iron, the expression of L-ferritin in the cortex of the Aβ-treated mice was upregulated compared with the control mice, while that of the Hamp + Aβ mice decreased substantially compared with Aβ-treated mice (Fig. 4c). In the hippocampus, L-ferritin showed an elevation in Aβ mice compared with the control mice and a reduction in Hamp + Aβ mice compared with Aβ mice (Fig. 4d). However, their differences did not reach statistical significance due to the wide variation of the values in the Aβ group.

We have also detected whether the alterations of brain iron affected the iron in neurons by double staining of H-ferritin with neuronal marker NeuN. As shown in Supplementary Fig. S4, the iron in the granular neurons of the hippocampus in Aβ-treated mice was substantially increased, whereas that of the Hamp + Aβ mice was reduced compared with Aβ mice, indicating the overexpression of hepcidin attenuated iron deposition in granular neurons of hippocampal CA3 regions. No significant difference in the iron levels of cortical neurons was observed between different groups of mice (Supplementary Fig. S4).

### Astrocyte hepcidin overexpression ameliorated Aβ-induced dysregulation of iron homeostasis

**Fig. 5:**
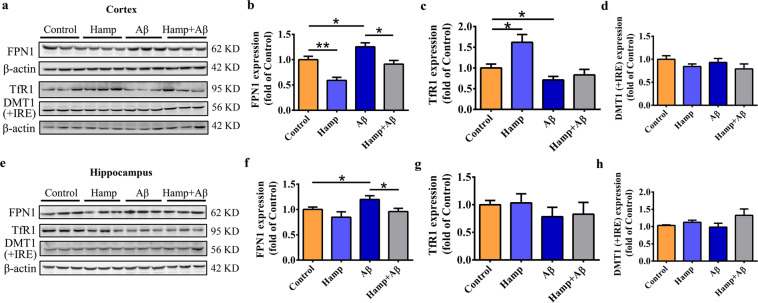
Astrocyte hepcidin overexpression prevented Aβ-induced dysregulation of iron homeostasis. Western blot was used to detect expression levels of FPN1, TfR1, and DMT1( + IRE) in the cortex (**a**–**d**) and hippocampus (**e**–**h**) of mice in different groups. Their relative expression levels in different groups as compared to the control group were calculated after normalizing each specific band to its respective β-actin band, and are presented as means ± SEM, *n* = 6. *P < 0.05 and **P < 0.01.

To assess the effects of astrocyte-overexpressed hepcidin on Aβ-induced dysregulation of brain iron metabolism, we determined the expressions of iron transporters, including iron efflux protein FPN1, uptake protein transferrin receptor 1 (TfR1), and divalent metal-ion transporter 1 (DMT1) with the iron-responsive element (+IRE)^44,45^. In the cortex, Aβ_25–35_ treatment caused a significant elevation in FPN1 expression in the Aβ group, and astrocyte hepcidin overexpression decreased FPN1 significantly in both the Hamp group and the Hamp + Aβ group (Fig. 5a, b). TfR1 decreased significantly in Aβ-treated mice, but was not affected much in Hamp + Aβ group compared with the control group (Fig. 5a, c). DMT1 (+IRE) did not show any statistically significant difference in all groups (Fig. 5a, d). In the hippocampus, the FPN1 expression in the Aβ group also showed an increase compared with the control group, and the Hamp + Aβ group showed a decrease in FPN1 expression compared with the Aβ group (Fig. 5e, f). The TfR1 and DMT1 (+IRE) expressions in the hippocampus of different groups of mice were not significantly changed (Fig. 5e, g, h). These observations indicated that Aβ stimulation led to alterations of cortical and hippocampal iron homeostasis, while hepcidin overexpression in astrocytes partly ameliorated its dysregulation.

### Astrocyte hepcidin-regulated iron influx at BBB

**Fig. 6:**
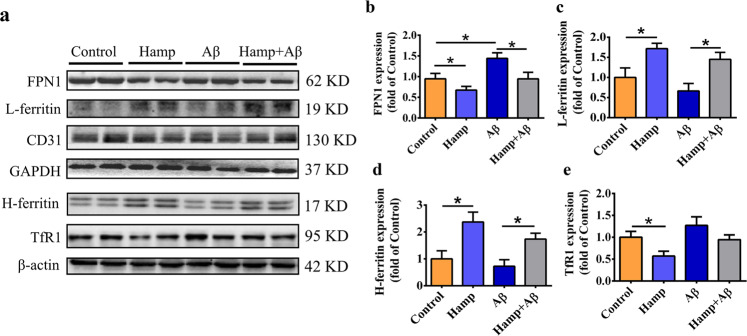
Astrocyte hepcidin overexpression reduced the iron influx from brain microvascular endothelial cells (BMVECs). BMVECs were isolated from cortical and hippocampal tissues. Western blot was used to detect expression levels of FPN1 (**a**, **b**), L-ferritin (**a**, **c**), H-ferritin (**a**, **d**), and TfR1 (**a**, **e**). CD31 was used as a specific marker for blood vessels, and GAPDH and β-actin were internal controls. The relative expression levels of FPN1 and L-ferritin in different groups as compared to the control group were calculated after normalizing each specific band to the CD31 band, the relative levels of H-ferritin and TfR1 were calculated after normalizing each specific band to their respective β-actin band. Data are presented as means ± SEM, *n* = 6. *P < 0.05 and **P < 0.01.

As indicated, hepcidin from astrocyte could interact with FPN1 on BMVECs^31,32^, which may influence the iron influx at BBB. We therefore explored whether the decrease in brain iron in Hamp + Aβ mice was achieved by inducing degradation of FPN1 on BMVECs. The BMVECs were isolated from brain tissues of mice of different groups, and their protein expression levels were determined by western blot. When treated with Aβ_25–35_, the levels of FPN1 and TfR1 were upregulated, indicating a higher iron release from blood to brain tissue (Fig. 6a–d). In contrast, the expression of FPN1 on BMVECs was decreased significantly in the Hamp group (Fig. 6a, b). The decrease of FPN1 reduced iron release from BMVECs and resulted in higher iron content in BMVECs, which was consistent with the upregulation of iron storage protein L-ferritin and H-ferritin (Fig. 6c, d). Therefore, in the Hamp + Aβ group, FPN1 significantly reduced as compared with the Aβ-treated group (Fig. 6a, b), reflecting a lower iron efflux from BMVECs into the brain tissue. These results imply that hepcidin overexpression in astrocytes could reduce brain iron uptake by inducing degradation of FPN1 on BMVECs, thereby attenuating iron deposition resulted from Aβ_25–35_ treatment.

### Astrocyte hepcidin attenuated Aβ-induced inflammation as well

**Fig. 7:**
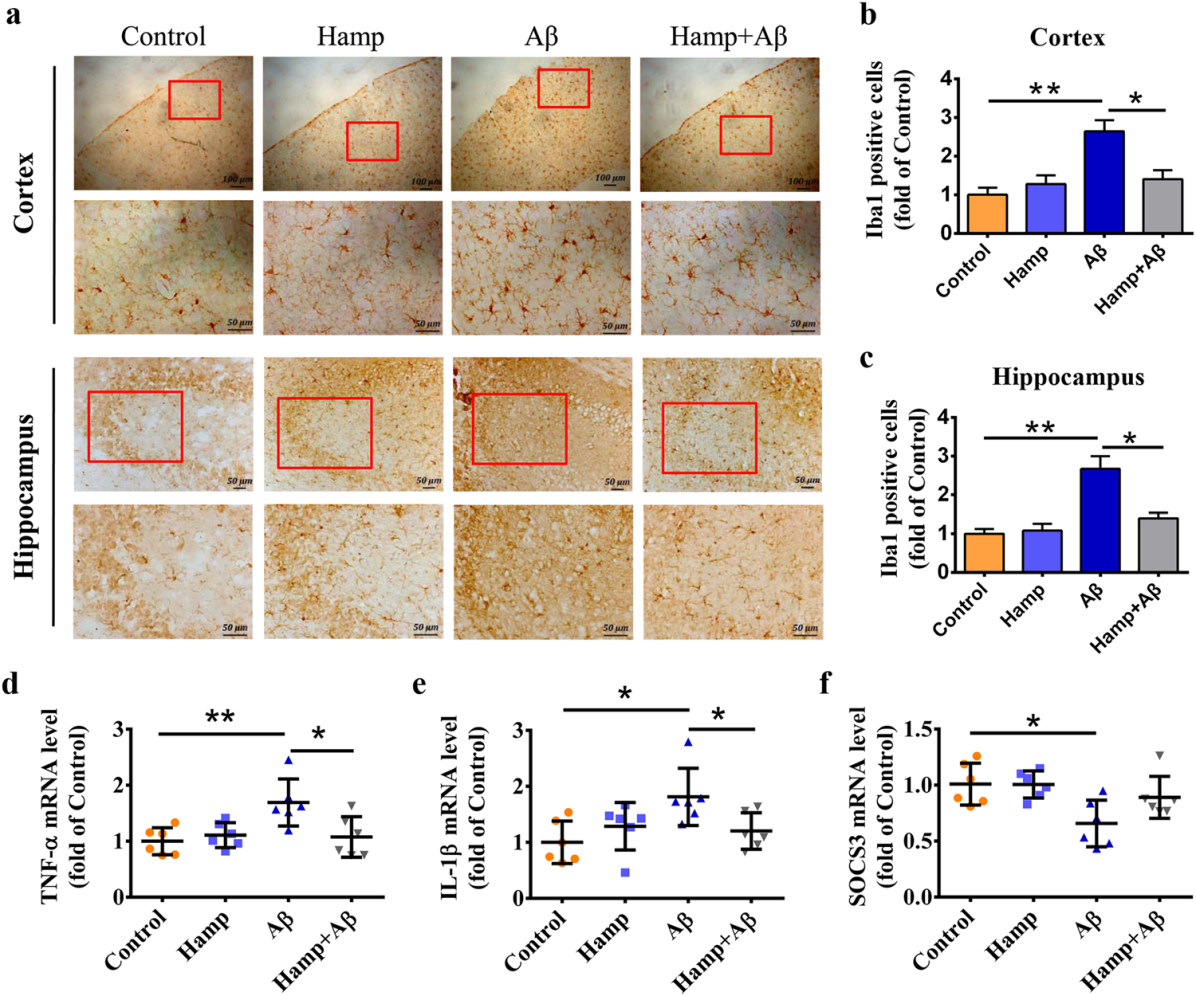
Astrocyte hepcidin attenuated Aβ-induced inflammation. **a** Immunohistochemical stainings of the microglia marker-Iba1 in the cortex and hippocampus were performed. Bar = 100 μm or 50 μm, as indicated. **b**, **c** The statistical analysis of relative Iba1-positive cell levels in cortical sections (**b**) and hippocampal sections (**c**). Data are presented as the means ± SEM, *n* = 6. **d**–**f** Detection of TNF-α (**d**), IL-1β (**e**), and SOCS-3 (**f**) mRNA levels in mouse cortex by qRT-PCR. Data are presented as the means ± SD, *n* = 6. *P < 0.05 and **P < 0.01.

It has been reported that Aβ can activate the microglia and increase the release of inflammatory factors, and hepcidin can prevent acute cytokine-induced inflammatory responses^46,47^. Therefore, we determined the levels of microglia and inflammatory factors in different groups of mice. The Iba1 immunohistological staining in the cortex and hippocampus revealed substantial changes in the number and morphology of microglia in mice injected with Aβ_25–35_ compared with the control group (Fig. 7a–c), whereas the Hamp + Aβ mice showed a decreased number of microglia compared with Aβ-injected mice (Fig. 7a–c). These inferred that hepcidin could suppress Aβ-induced microglial activation. Consistent with this, the inflammatory cytokines, tumor necrosis factor α (TNF-α) and interleukin 1β (IL-1β), were upregulated in the cortex of Aβ-injected mice compared with the control mice (Fig. 7d, e), whereas those of the Hamp + Aβ mice remained steady. On the other hand, the suppressor of cytokine signaling 3 (SOCS-3) was largely reduced in the Aβ-injected mice, but its level in Hamp + Aβ mice did not decrease significantly compared with the control mice (Fig. 7f). These indicated that astrocyte hepcidin effectively suppressed Aβ-induced inflammation.

## Discussion

It has been reported that increasing hepcidin expression or treatment with hepcidin peptide could reduce brain iron and protect neurons from iron-mediated oxidative damage^26,27^, and the expression and distribution of hepcidin in the brain were closely depended on age and inflammatory status^28–30^. Besides, hepcidin secreted by astrocytes was proposed to control iron intake at BBB by regulating FPN1 on BMVECs^31,32^. These imply that modulating the level of hepcidin in the brain, especially hepcidin expressed by astrocytes, might be a promising therapy opportunity for iron-overload brain disorder, including AD. In this study, in vivo evidences were collected to show whether increasing the expression of astrocyte hepcidin could reduce brain iron level and subsequently relieve AD symptoms.

We successfully overexpressed hepcidin in astrocytes of mice using a modified pAAV plasmid with astrocyte-specific GFAP promoter and then injected mice with Aβ_25–35_ to induce injury. Behavioral experiments revealed that the elevated astrocyte hepcidin partially protected spatial learning and memory impairment in mice induced by neurotoxic Aβ_25–35_, which possibly involved its protection on synaptic function because the PSD-95 expression was not reduced significantly in Hamp + Aβ mice. From the stained tissue sections of the cortex and hippocampus, largely decreased numbers of apoptotic cells were observed in astrocyte hepcidin overexpressing mice. The levels of ROS and 4-HNE were also reduced. By assessing iron metabolism, we found that astrocyte hepcidin overexpression indeed decreased Aβ-induced brain iron overload. Besides, the Aβ-induced iron dyshomeostasis, reflected by the alterations of various iron transporters, was also alleviated by the overexpressed astrocyte hepcidin. These all implied that the overexpression of hepcidin in astrocytes protected mice from Aβ-induced cognitive decline, cell apoptosis, and oxidative damage in the brain, which possibly linked to its regulation of brain iron accumulation.

The assessment of the FPN1 level on BMVECs showed that FPN1 expression was significantly decreased by the elevated astrocyte hepcidin. Consistent with this, L-ferritin in BMVECs of Hamp + Aβ mice markedly increased, reflecting an intracellular iron accumulation status of BMVECs. Due to the activation of the cellular iron regulatory systems that initiated by the interaction of iron regulatory proteins (IRPs) with IREs in responses to a higher iron load^13,48^, ferritin expression would increase, but TfR1 should decrease in the Hamp + Aβ mice to lower the iron intake. However, no statistically significant decrease in TfR1 was observed, which likely due to the wide variation of the values in Hamp + Aβ group. These results inferred that the overexpressed astrocyte hepcidin influenced iron uptake at BBB and decreased brain iron load, thereby protecting against Aβ-induced injury.

Previous studies by Urrutia et al. have found that hepcidin peptide could attenuate Aβ-induced inflammatory and pro-oxidative responses in astrocytes and microglia, alleviating oxidative damage and neurotoxicity^47^. However, the involvement of hepcidin’s regulation of iron metabolism in these inhibitory effects was not investigated. Our results showed that overexpression of astrocyte hepcidin in mice could also inhibit Aβ-induced microglial activation and block inflammatory cytokine release. This protective effect could be primarily attributed to the function of hepcidin in reducing brain iron load, and could also be partially due to its inhibition on cytokines TNF-α and IL-1β and its activation on SOCS-3^46,49^.

As realized of the limitation on drug diffusion range of ICV injection, we additionally performed hippocampal injections of hepcidin and Aβ_25–35_, to fully evaluate the protective effects of hepcidin. As shown in Supplementary Fig. S5, the hippocampal injection of Aβ_25–35_ led to severe apoptosis in the dentate gyrus of the hippocampus, and also induced the increase of 4-HNE-positive cells and microglia. When hepcidin was overexpressed in astrocytes, these cell damage and inflammatory responses caused by Aβ_25–35_ were remarkably alleviated. These results were consistent with the result of ICV injection, which further confirmed the protective effects of astrocyte hepcidin on hippocampal damage.

It was noted that the role of hepcidin in neuronal iron load demonstrated by different studies were contradictory. In contrast to the beneficial effects revealed by Urrutia and Zhou et al.^26,27,47^, researchers found that a high level of hepcidin could be destructive^25,50^. The increased hepcidin produced by astrocytes and microglia directly acted on FPN1 of neurons, leading to a reduction in iron export from neurons, which aggravated neuronal oxidative damage^29,51,52^. Recently, Vela proposed a dual role of hepcidin in the brain, suggesting whether there is inflammation or not is the main reason for this contradiction^25^. He hypothesized that pretreatment with hepcidin could protect brain cells from oxidative damage, while hepcidin induction during inflammation would increase iron-dependent injury^25^. In our study, the overexpression of hepcidin in astrocyte was a pretreatment factor, and a protective effect did occur. Whether the beneficial role of astrocyte hepcidin can sustain with increasing levels of inflammation requires more in-depth research.

**Fig. 8:**
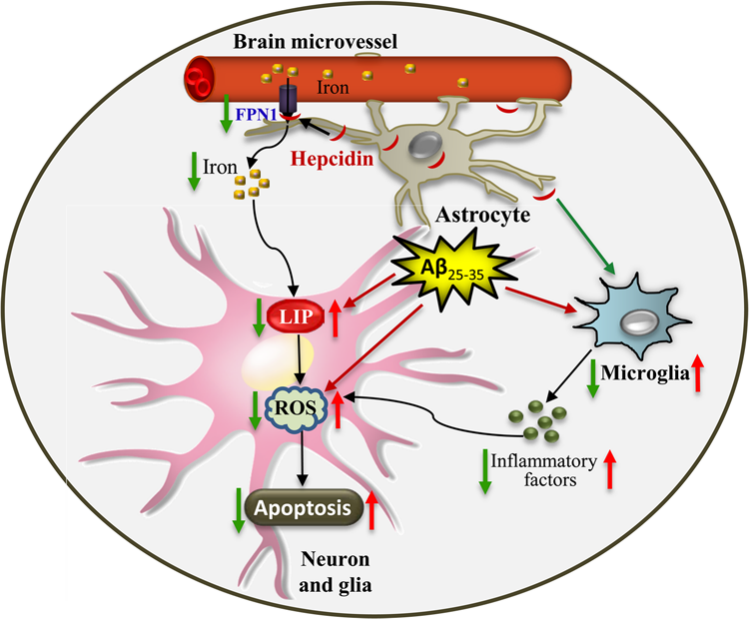
A schematic representation of the protective role of astrocyte hepcidin on Aβ-induced apoptosis. Aβ treatment results in higher intracellular LIP, which can induce oxidative stress and inflammation, leading to neuronal death. When hepcidin secreted by astrocytes is upregulated, it interacts with FPN1 on brain microvascular endothelial cells (BMVECs) and reduces brain iron intake, thereby ameliorating LIP elevation caused by Aβ. This in turn attenuates iron-dependent reactive oxygen species (ROS) production. Besides, the overexpressed hepcidin also inhibits the activation of microglia induced by Aβ, and blocks the release of pro-inflammatory factors. Ultimately the apoptosis induced by Aβ is largely prevented.

In summary, as shown in Fig. 8, we proposed that the hepcidin overexpressed by astrocytes primarily reduced FPN1 of BMVECs, thereby reduced iron efflux from BMVECs and lowered brain iron content. The high level of the cellular liable iron pool (LIP) caused by Aβ was then ameliorated^53^. This in turn attenuated iron-dependent oxidative damage, and ultimately suppressed the apoptosis induced by Aβ via MAPK signals in neurons and glial cells of the cortex and hippocampus. Besides, the overexpressed hepcidin also inhibited the Aβ-induced activation of microglia, blocked the release of pro-inflammatory factors, and induced the increase of cytokine suppressors. These effectively prevented the aggravation of apoptosis and improved the behavioral performance of mice. Our study, for the first time, demonstrated with in vivo evidence that astrocyte-specific overexpression of hepcidin altered brain iron metabolism, and protected against Aβ-induced oxidative damage in mice, which provides more insights into the therapy of iron overloaded-related brain disorders.

## Materials and methods

### Animals

Three-month-old male Balb/C mice were obtained from Hebei Medical University. Mice were housed under conditions controlled for temperature (22 °C) and humidity (40%), using a 12 h/12 h light/dark cycle. Mice were fed a standard rodent diet and water ad libitum. All procedures were carried out in accordance with the National Institutes of Health Guide for the Care and Use of Laboratory Animals, and were approved by the Animal Ethics Committee of Hebei Normal University.

### Plasmid construction

To clone the astrocyte-specific expression plasmid of hepcidin (*Hamp*), pAAV-EGFP plasmid was used as a vector backbone. The promoter sequence of the glial fibrillary acidic protein (GFAP) was amplified by PCR^54^, and inserted into the pAAV-EGFP plasmid to replace its CMV promoter. The full-length cds of the mouse *Hamp* gene were amplified and inserted into the MCS and replaced the EGFP gene. The β-globin intron was also removed. This expression vector was named as pAAV-gfap:*Hamp*. The pAAV-gfap backbone vector without inserting *Hamp* was used as control. The primers used to amplify GFAP promoter and *Hamp* gene were as follows:

GFAP promoter forward: 5′-CCGACGCGTGAGCTCCCACCTCCCTCTCTG-3′

GFAP promoter reverse: 5′-CCGGAATTCTCACCTGCTCTGGCTCTGCTCGCT-3′

*Hamp* forward: 5′-CCGGAATTCATGGCACTCAGCACTCGGACC-3′

*Hamp* reverse: 5′-CCGCTCGAGCTATGTTTTGCAACAGATACCACACTG-3′.

### Antibodies

The following antibodies were used: β-actin antibody (cw0096m, CWbio, China), FPN1 antibody (MTP11-S, ADI, USA), DMT1 (+IRE) antibody (NRAMP21-S, ADI, USA), 4-HNE antibody (HNE-11S, ADI, USA), TfR1 antibody (ab84036, Abcam, USA), L-ferritin antibody (ab109373, Abcam, USA), H-ferritin antibody (ab183781, Abcam, USA), hepcidin antibody (ab30760, Abcam, USA), Aβ_22–35_ antibody (A3356, Sigma, USA), phospho-p38 (p-p38) antibody (4511S, CST, USA), p38 antibody (8690S, CST, USA), phospho-ERK (p-ERK) antibody (9102S, CST, USA), ERK antibody (4372S, CST, USA), Bcl-2 antibody (12789-1-AP, Proteintech, China), Bax antibody (50599-2-Ig, Proteintech, China), GFAP antibody (MAB360, Millipore, USA), Iba1 antibody (MABN92, Millipore, USA), NeuN (ab104224, Abcam, USA), CD31 (77699T, CST, USA), PSD-95 (ab18258, Abcam, USA), anti-rabbit IgG (RPN4301, Amersham, UK), anti-mouse IgG (RPN4201, Amersham, UK), DyLight 488 goat anti-mouse IgG (A23210, Abbkine, USA), DyLight 549 goat anti-rabbit IgG (A23320, Abbkine, USA), and DyLight 549 goat anti-mouse IgG (A23310, Abbkine, USA).

### Plasmid injection, electroporation, and Aβ treatment

Mice, weighing 22–25 g, were randomly divided into four groups, nine mice per group for behavior tests or six mice per group for the rest of other assays according to the previous experiments^36^. Two groups were injected with pAAV-gfap:*Hamp* (1.5 μg/μl, 4 μl) via ICV injection into the right lateral ventricle (0.5 mm posterior and 1.0 mm lateral to the bregma, 2.5 mm below the surface of skull) or intrahippocampal injection into the right hippocampus as specifically stated (1.9 mm posterior and 1.0 mm lateral to the bregma, 1.5 mm below the surface of skull). The other two groups were injected with vector plasmid control. Following injection, the brains were electroporated with square wave pulses from an Electro Square Porator ECM830 (BTX Inc, San Diego, CA). The tweezer-like electrodes, 6–8 mm apart, were placed on the mouse skull across the injection site, with a positive electrode on the right side of the mouse skull such that the DNA was driven into the cells on right side of the brain. The brains were pulsed three times for 50 ms each at 125 V at 1 s intervals.

Aβ_25–35_ peptide (A4559, Sigma, USA) or Aβ_35–25_ peptide (A2201, Sigma, USA) was dissolved in sterile saline and aggregated by incubation at 37 °C for 4 days before use as previously described^36^. After 48 h of Hamp plasmid injection, the aggregated form of Aβ_25–35_ was administrated (7.5 nmol in 5 μl saline) into one of the *Hamp* plasmids injected group and one of the control group. The rest two groups were injected with saline as controls. The four groups were named as control (vector plasmid + saline), Hamp (*Hamp* plasmid + saline), Aβ (vector + Aβ_25–35_), and Hamp + Aβ (*Hamp* plasmid + Aβ_25–35_). After injection, mice were housed for 7 days under normal conditions. Mice were trained and tested in a Morris water maze (MWM), and then killed under anesthesia by transcardiac perfusion. The brains were immediately collected and subjected to further analyses, including tissue section, western blot, etc. All the experiments were repeated at least three times. Blinding was not performed in the analysis.

### Behavioral test by MWM

Spatial learning and memory deficits were assessed using the MWM test^36^. A visible round platform (diameter = 9 cm) was placed in the water maze tank during the training period. For the hidden platform test, the platform was placed 1 cm below the water surface at the midpoint of the fourth quadrant. All mice were performed with four trials per day for 5 days, with the escape platform kept at a constant position. Each trial lasted for 120 s or ended as soon as the mice reached the submerged platform. The “escape latency”, the time to reach the platform in the water maze, was recorded. At the 6th day, a probe test was performed after removing the platform, using the metric “time spent in the target quadrant” to investigate the maintenance of memory.

### Quantitative real-time reverse transcription-PCR (qRT-PCR)

The total RNA from cortical or hippocampal tissue was extracted by TRIzol agent (Invitrogen, China). Total RNA was reverse-transcribed with MMLV reverse transcriptase (TaKaRa Biotechnology, China) and Oligo-dT primers (TianGen Biotechnology, China). The SYBR green PCR Master Mix (Beijing ComWin Biotechnology, China) was used for PCR amplification. The cycle time (Ct) values for the gene of interest were first normalized with β-actin in the same sample, and then the relative differences between the control and each of the other groups were calculated using the equation 2^-ΔΔCt^, and expressed as relative fold changes of the control group. The expression of hepcidin mRNA and inflammatory factors mRNA (TNF-α、IL-1β and SOCS-3) were determined. The primer sequences used for amplification were as follows:

β-actin forward: 5′-AGGCCCAGAGCAAGAGAGGTA-3′

β-actin reverse: 5′-TCTCCATGTCGTCCCAGTTG-3′

Hepcidin forward: 5′-AGACATTGCGATACCAATGCA-3′

Hepcidin reverse: 5′-GCAACAGATACCACACTGGGAA-3′

TNF-α forward: 5′-AGGCGGTGCCTATGTCYCA-3′

TNF-α reverse: 5′-GAGGCCATTTGGGAACTTCT-3′

IL-1β forward: 5′-GAAATGCCACCTTTTGACAGTG-3′

IL-1β reverse: 5′-CTGGATGCTCTCATCAGGACA-3′

SOCS-3 forward: 5′-GTGGAGAGGCTGAGGGACTC-3′

SOCS-3 reverse: 5′-GGCTGACATTCCCAGTGCTC-3′

### Assessment of apoptosis

The mouse brains were postfixed with 4% paraformaldehyde in 0.1 M phosphate buffer. Serial coronal sections were cut at 15-μm thick on a freezing microtome (Leica CM1950, Leica Microsystems, China) and mounted onto slides. The presence of apoptosis in the mouse cortex and hippocampus was assessed by the terminal deoxynucleotidyl transferase-mediated FITC-12-dUTP nick-end labeling method (TUNEL) following the manufacturer’s protocol (TUNEL BrightGreen Apoptosis Detection Kit, Vazyme, China). Nuclei were counterstained with DAPI. For each group, sections from three different mice were stained, and the numbers of TUNEL-DAPI-positive cells located at similar positions in all groups were quantified for statistical analysis.

### Western blot analysis

Tissues of the cortex and hippocampus were homogenized and sonicated in RIPA buffer containing 1% NP40 and protease inhibitor cocktail tablets (Roche Diagnostics GmbH, Roche Applied Science, Germany). After centrifugation at 12,000 × *g* for 20 min at 4 °C, the supernatant was collected, and protein concentration was measured using BCA Protein Quantification Kit (Yeasen Biotechnology, China). In total, 30 μg of protein from each sample was resolved by SDS-PAGE on 12% or 10% gels and then transferred to NC membranes. The membranes were blocked in 5% non-fat milk TBS-T buffer (20 mM Tris-HCl, 137 mM NaCl, and 0.1% tween-20, pH 7.6) for 1.5 h at room temperature, followed by incubation with primary antibody overnight at 4 °C. After washing four times with TBS-T, the membranes were then incubated with HRP-conjugated secondary antibody for 1.5 h at room temperature. Immunoreactive proteins were detected using the enhanced chemiluminescence method and quantified by transmittance densitometry (FUJIFILM Corporation, Tokyo, Japan).

### Immunohistochemistry and immunofluorescence

For immunohistochemical staining, sections of the cortex and hippocampus were immersed in methanol with 3% hydrogen peroxide (H_2_O_2_) for 20 min to reduce endogenous peroxidase activity. Antigen retrieval was performed in a microwave oven for 10 min in 10 mM citrate buffer (pH 6.0). After blocking for 1 h with normal goat serum, the slices were incubated overnight at 4 °C with mouse anti-Iba1 monoclonal antibody (1:500). The sections were then incubated with biotinylated goat anti-mouse serum for 1 h at 37 °C. Thereafter, sections were treated with avidin-biotinylated horseradish peroxidase complex (Zymed Laboratories, 1:200) for 1 h at 37 °C and then stained using a 3, 3′-diaminobenzidine tetrahydrochloride (DAB) kit (Zsbio, China). After staining, the sections were rinsed with tap water for 20 min to block the reaction, followed by the gradient dehydration with alcohol, and then were transparentized by xylene and sealed by resin adhesive.

For the immunofluorescence assay, after blocking for 1 h with goat serum, the slices were incubated overnight at 4 °C with mouse anti-GFAP monoclonal antibody (1:500), rabbit anti-hepcidin antibody (1:300), or rabbit anti-4-HNE antibody (1:400). The slices were washed three times with 0.01 M PBS for 5 min. Secondary antibodies DyLight 488 AffiniPure Goat Anti-Mouse IgG or DyLight 549 AffiniPure Goat Anti-Rabbit IgG were then used in incubation for 1 h at 37 °C. Finally, after washing and mounting, the sections were photographed. Negative controls were processed using the same procedures, but the primary antibody was omitted. All sections were visualized and quantified under either an upright fluorescence microscope (ZEISS Axio Imager, Germany) or a fluorescence confocal microscope (Olympus FV3000, Japan). For each group, quantification was performed in sections from three different mice.

### Measurement of ROS

ROS levels were quantified by measuring the fluorescence of 2′,7′-dichlorofluorescein diacetate (DCFH-DA) using a commercial kit (Nanjing Institute of Bioengineering, Nanjing, China) according to the manufacturer’s instructions^55^. First, the dissected cortical or hippocampal tissues were placed in 0.01 M pre-cooled PBS, and then cut into pieces with medical scissors and digested with trypsin at 37 °C for 10 min. The formulation was then filtered using a 200-μm membrane filter and centrifuged at 500 × *g* for 10 min. The cell pellet was resuspended and diluted with PBS to a final concentration of 5 × 10^6^ cells/ml. The cells were incubated with 10 μM DCFH-DA for 50 min at 37 °C in the dark, and then centrifuged at 1000 × *g* for 10 min at 4 °C. The pellet was washed twice with PBS and resuspended in PBS. Fluorescence intensity was detected by a multi-function microplate reader (Synergy H4, BioTek, VT, USA) at an optimal excitation wavelength of 485 nm and an emission wavelength of 525 nm.

### Synchrotron radiation micro X-Ray fluorescence (μ-XRF)

The fixed brain was subjected to gradient dehydration in 10, 20, and 30% sucrose solutions, and then frozen and sliced to a thickness of 50 μm, and attached to a 3-mm thick Mylar film. The brain slices were dried and placed in a vacuum desiccator. At the 4W1B line station of the Beijing Synchrotron Radiation Facility (BSRF) center, X-ray fluorescence spectrometer was used to measure the distribution of iron elements in the slices. The range is from 150 mA to 250 mA, and the electron energy in the storage ring is 2.2 GeV. The size of the X-ray spot is 50 × 50 μm. The energy of the incident X-ray is monochromated at 15 keV by a W/B4C high photon flux dual-layer film single-frequency device, and is focused by a multi-capillary lens to a diameter of 50 μm. The live time at each point is 25 s, with a step size of 100 μm. The scan area for the cortical region is 2000 × 1000 μm, and for the hippocampal region is 2000 × 1200 μm. Data conversion was performed by PyMca software and plotted with Origin 8.0.

### Isolation of BMVECs from mouse brain

The anesthetized mice were perfused with pre-cooled 0.9% saline, and BMVECs from the tissues of the cerebral cortex and hippocampus were isolated as reported with small modifications^56,57^. The separated tissues were placed in a pre-cooled homogenizer after weighting. Pre-cooled HBSS buffer (HEPES 10 mM, NaCl 141 mM, KCl 4 mM, MgSO_4_•3H_2_O 1 mM, NaH_2_PO_4_•2H_2_O 1 mM, CaCl_2_ 2.5 mM, glucose 10 mM, and sodium pyruvate 1 mM) was added at the ratio of tissue to HBSS (mass: volume) of 1:3. After homogenization, the mixture was transferred to a 1.5-ml EP tube. An equal volume of 32% dextran solution was then added, fully mixed, and centrifuged at 7245×*g* for 15 min at 4 °C. The supernatant and the off-white brain tissues were removed. The brain microvessels at the bottom of the centrifuge tube were washed twice with 16% dextran in HBSS buffer and then twice with HBSS buffer. After centrifugation, the pellet was resuspended in 500 μl of HBSS buffer, and then filtered through 100-μm membrane filters. After another centrifugation, the pellet was added with RIPA buffer containing 1% NP40 and protease inhibitor cocktail tablets and kept on ice for 30 min with vortexing for 15 s in every 10 min. After centrifugation at 12,000×*g* for 20 min at 4 °C, the supernatant was collected, and protein expression was measured by western blot.

### Statistical analysis

The data were expressed as the means ± SD or means ± SEM, as specifically indicated in the figure legends. Datasets were tested for normality distribution, and those passed the normality test were examined by one-way analysis of variance (ANOVA) with Tukey’s post hoc tests by SPSS 21.0 software. *P* values of < 0.05 were considered to be statistically significant; *P* < 0.01 was considered to be remarkably significant.

## Supplementary information

Supplementary Figure legends

Supplementary Figure S1

Supplementary Figure S2

Supplementary Figure S3

Supplementary Figure S4

Supplementary Figure S5
