# Supplementary material for: Hepcidin overexpression in astrocytes alters brain iron metabolism and protects against amyloid-β induced brain damage in mice
Source: Cell Death Discov. 2020 Oct 30;6:113. doi: 10.1038/s41420-020-00346-3 (PMC7603348; doi:10.1038/s41420-020-00346-3)
Supplement: Supplementary file 1 — Supplementary Figure legends [file 41420_2020_346_MOESM1_ESM.docx]

**Supplementary Figure Legends**

**Supplementary Fig. S1. Astrocyte hepcidin attenuated the Aβ-induced reduction in PSD-95.** (a) Quantifications for PSD-95 mRNA in hippocampus of mouse brain by qRT-PCR. Data are presented as the mean ± SD, n = 6. *P < 0.05. (b) Western blot was used to detect PSD-95 protein in hippocampus of mouse brain. The expression level was normalized to β-actin and expressed as the mean ± SEM. n = 6. *P < 0.05 and **P < 0.01.

**Supplementary Fig. S2. Aβ_25-35_-induced apoptosis in both neurons and glia cells.** (a) Mice were ICV-injected with Aβ_25-35_ or Aβ_35-25_ (control). Dectection of the apoptotic cells in the cortical section was carried out by TUNEL staining and then double stained with anti-NeuN antibody. (b) IF staining with anti-Aβ_22-35_ antibody to confirm the levels of Aβ in mouse brain. The arrows indicate the locations of Aβ aggregates.

**Supplementary Fig. S3. Astrocyte hepcidin suppressed Aβ-induced MAPK/p38/ERK phosphorylation.** Western blot was used to detect the phosphorylation of p38 and ERK proteins in cortex (a-c) and hippocampus (d-f) of mouse brain. The ratios of p-p38/p38 and p-ERK/ERK were calculated and expressed as fold of the Control group. Data are presented as the means ± SEM, n = 6. *P < 0.05 and **P < 0.01.

**Supplementary Fig. S4. Astrocyte hepcidin attenuated Aβ-induced iron deposition in neuronal cells.** Immunofluorescence staining of NeuN (green) and FTH (red) was carried out in sections of cortex (a) and hippocampus (b). DAPI was used for nuclear staining. Bar = 20 μm (cortex) or 50 μm (hippocampus).

**Supplementary Fig. S5. Astrocyte hepcidin overexpression by hippocampal injection also showed a protective effect on apoptosis, oxidative damage and inflammation induced by hippocampal injected Aβ.** (a) TUNEL-positive cells and DAPI-stained cell nuclei of the DG region in the hippocampus of mouse brains. (b) Immunofluorescence staining against the 4-HNE (red) was performed in hippocampus. DAPI (blue) was used for nuclear staining. (c) Immunohistochemistry staining against the microglia marker-Iba1 in the hippocampus.
