# Supplementary figures and images for: Hepcidin overexpression in astrocytes alters brain iron metabolism and protects against amyloid-β induced brain damage in mice
Source: Cell Death Discov. 2020 Oct 30;6:113. doi: 10.1038/s41420-020-00346-3 (PMC7603348; doi:10.1038/s41420-020-00346-3)

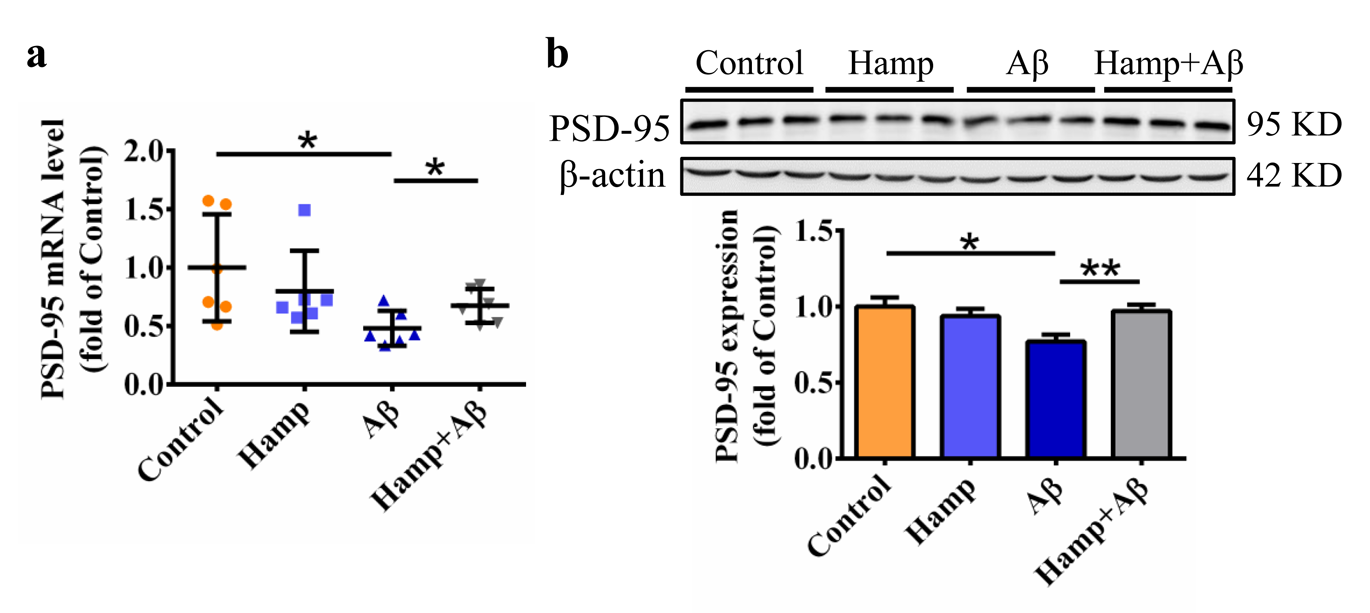

Supplement: Supplementary file 2 — Supplementary Figure S1 [file 41420_2020_346_MOESM2_ESM.tif]

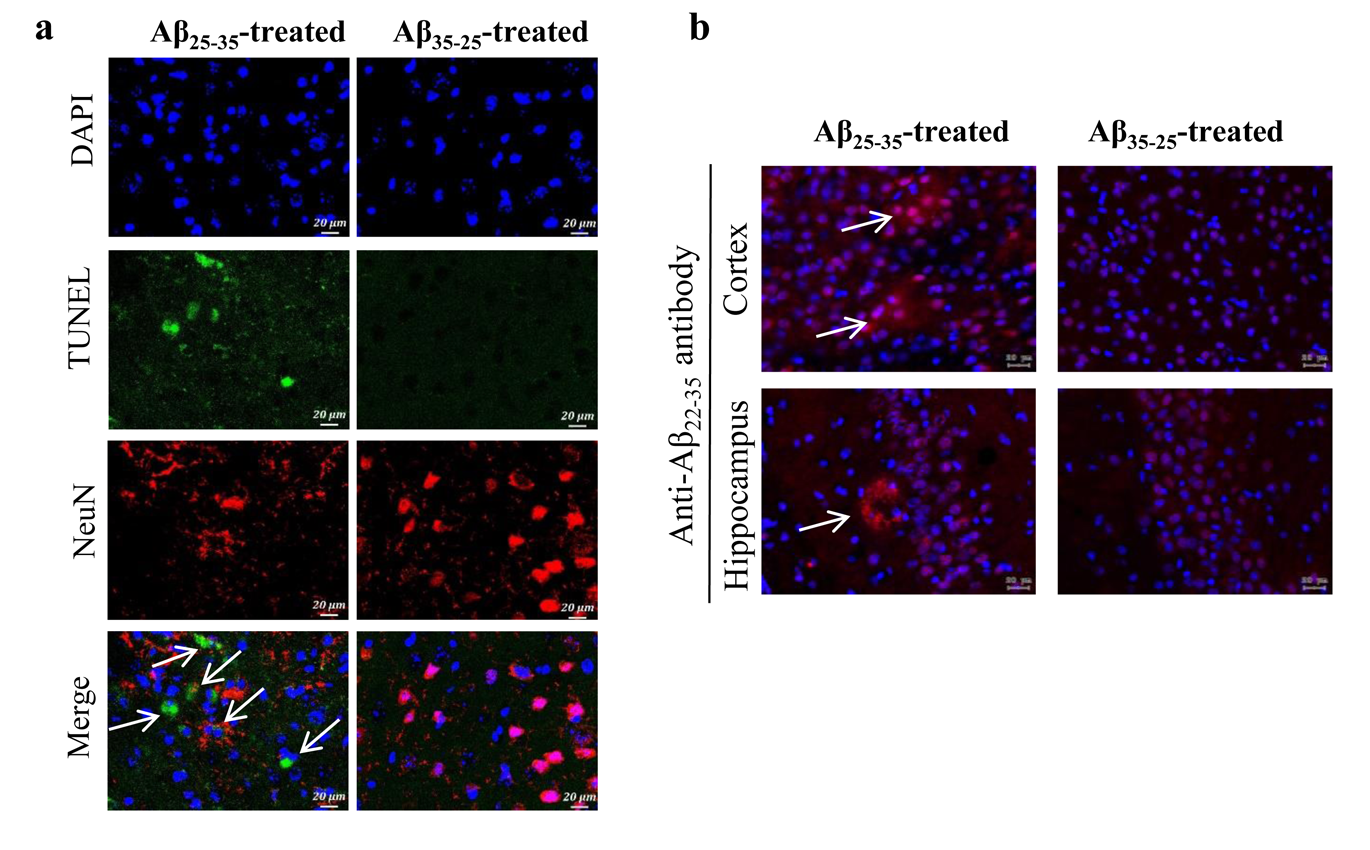

Supplement: Supplementary file 3 — Supplementary Figure S2 [file 41420_2020_346_MOESM3_ESM.tif]

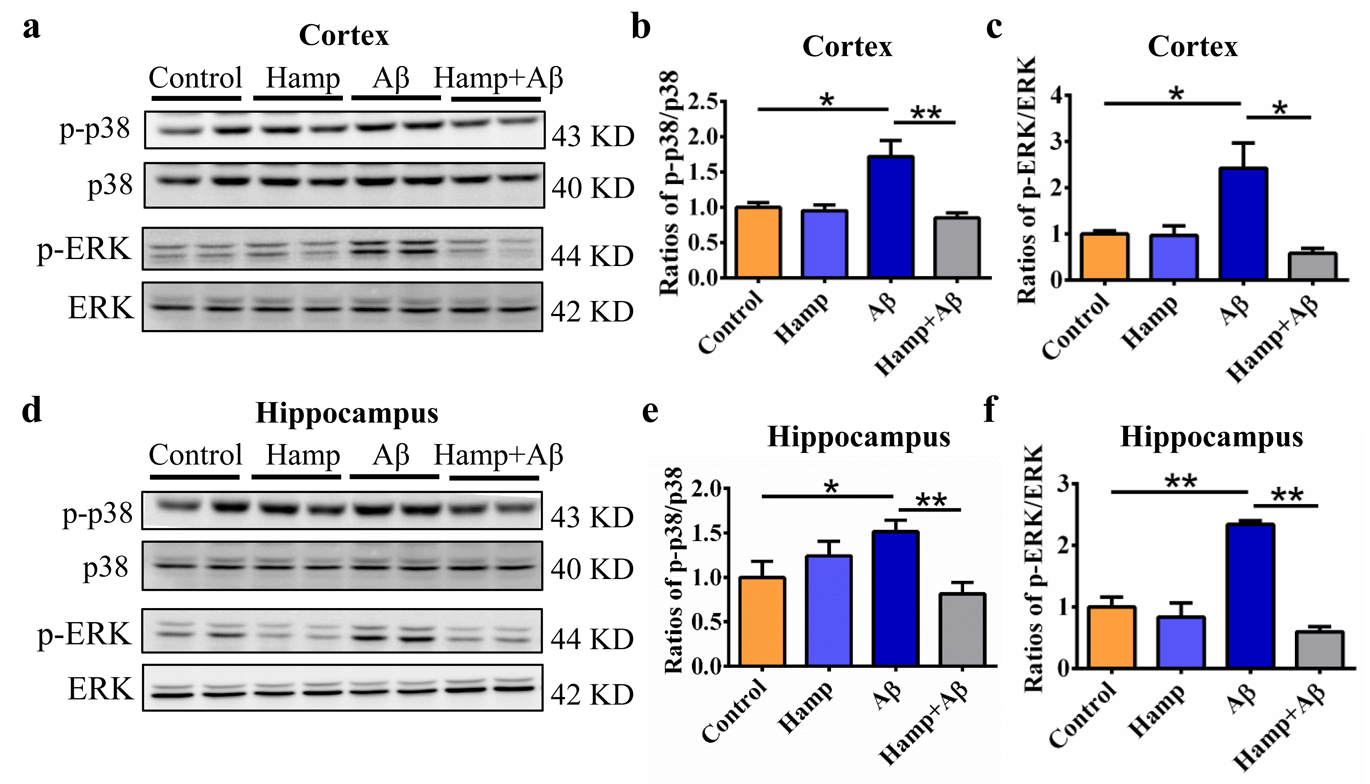

Supplement: Supplementary file 4 — Supplementary Figure S3 [file 41420_2020_346_MOESM4_ESM.tif]

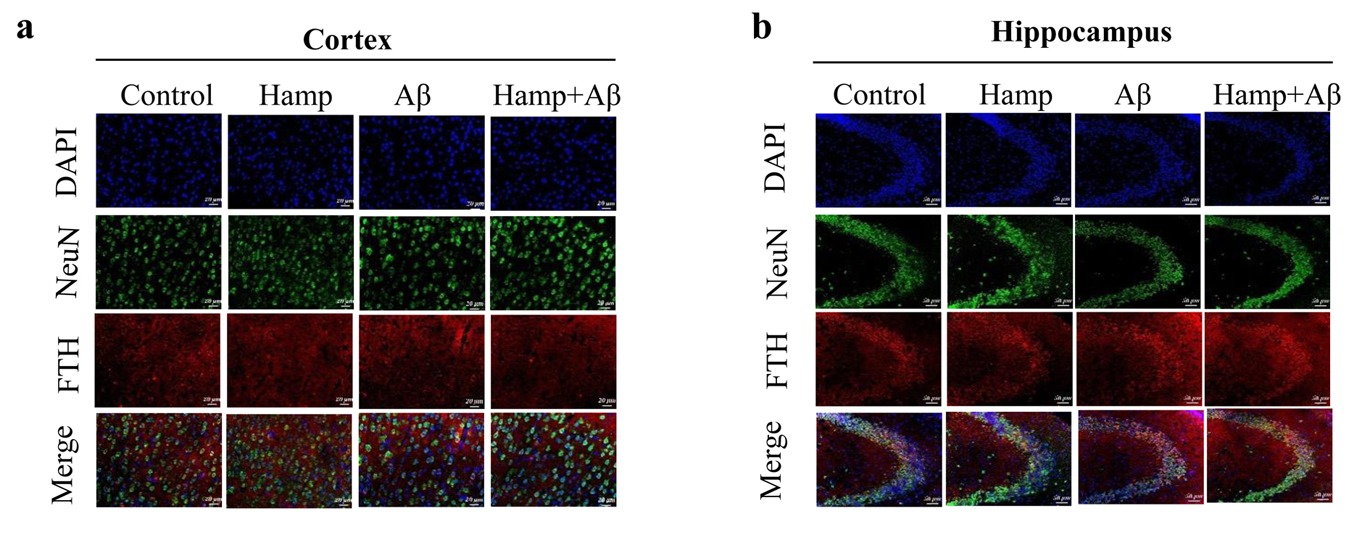

Supplement: Supplementary file 5 — Supplementary Figure S4 [file 41420_2020_346_MOESM5_ESM.tif]

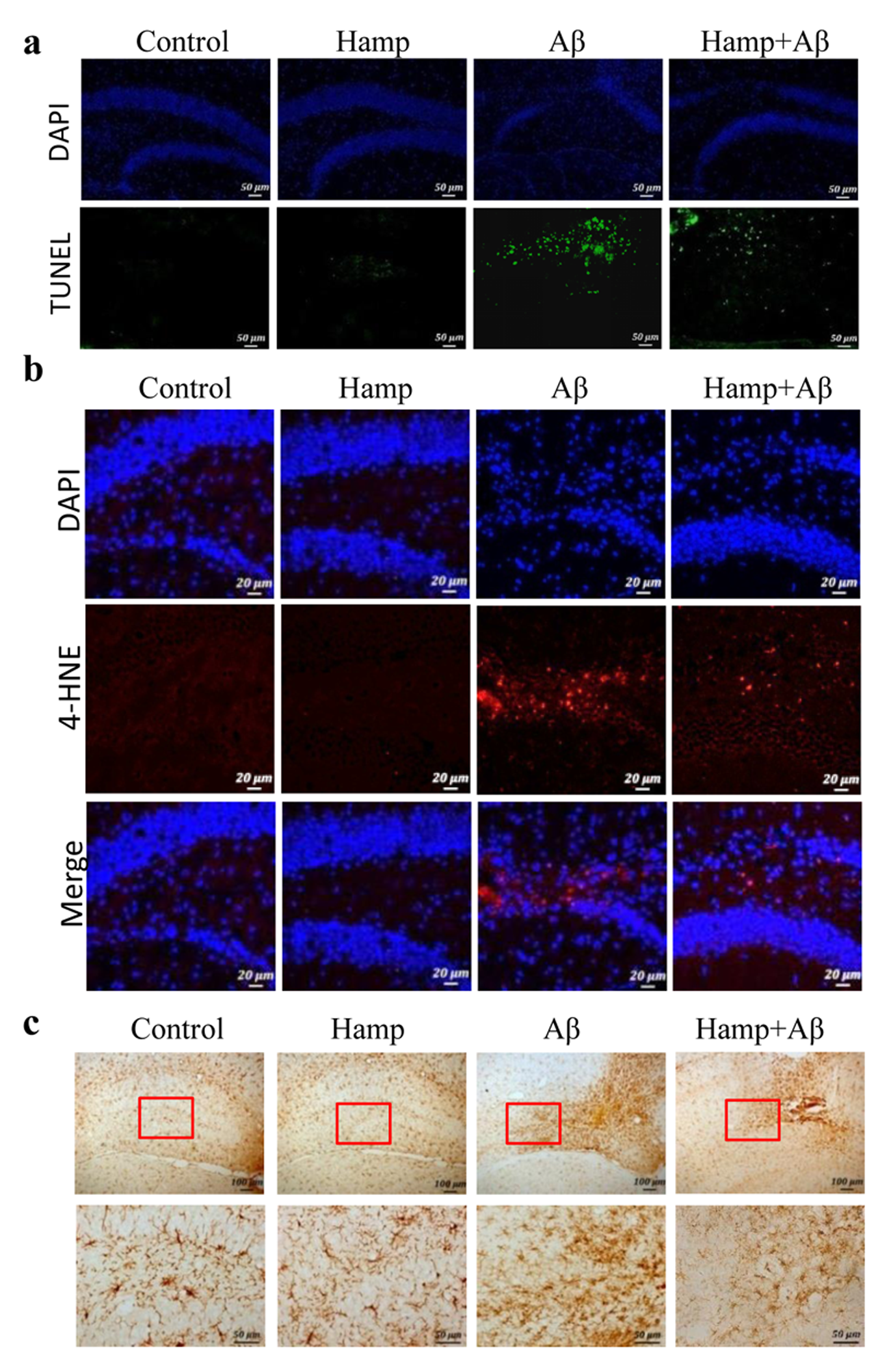

Supplement: Supplementary file 6 — Supplementary Figure S5 [file 41420_2020_346_MOESM6_ESM.tif]
